# Pim1 promotes IFN-β production by interacting with IRF3

**DOI:** 10.1038/s12276-022-00893-y

**Published:** 2022-11-29

**Authors:** Ryeojin Ko, Jeongin Seo, Hana Park, Nawon Lee, Soo Young Lee

**Affiliations:** 1 Department of Life Science, Ewha Womans University, Seoul, Korea; 2 The Research Center for Cellular Homeostasis, Ewha Womans University, Seoul, Korea

**Keywords:** Toll-like receptors, Phagocytes

## Abstract

The Pim (proviral integration site for Moloney murine leukemia virus) proteins compose a serine threonine kinase family whose members regulate cell proliferation, migration and cell survival. However, whether Pim kinases participate in innate immune responses is unclear. Here, we show for the first time that Pim1 plays an essential role in the production of interferon (IFN)-β by macrophages after their Toll-like receptor (TLR) pathway is activated by pathogen-associated molecular patterns (PAMPs). Specifically, Pim1 was quickly upregulated in an NF-κB-dependent manner after TLR stimulation with PAMPs. Pim1 deficiency reduced TLR3- or TLR4-stimulated IFN-β and IFN-stimulated gene (ISG) expression but not proinflammatory cytokine expression in macrophages. Mechanistically, Pim1 specifically upregulates IRF3 phosphorylation and nuclear translocation. However, this role is not dependent on Pim1 kinase activity. Rather, Pim1 appears to promote IRF3 phosphorylation by enhancing the formation of IFN-β signaling complexes composed of TRIF, TRAF3, TBK1, and IRF3. Poly (I:C)-treated *Pim1*^*−/−*^ mice produced less serum IFN-β and were less likely to survive than wild-type mice. These findings show for the first time that Pim1 participates in TLR-mediated IFN-β production, thus revealing a novel target for controlling antiviral innate immune responses.

## Immunology: A player in the front-line antiviral response

A signaling protein that promotes cell survival also helps manage the immune system’s ‘front-line’ response to viral infection. Immediately after infection, the cells of our innate or non-specific immune system rapidly mobilize to keep pathogens at bay until our T and B cells elicit a specific immune reaction. Soo Young Lee and colleagues at Ewha Womans University in Seoul, South Korea, have now linked the antiviral innate immune response to Pim1, a signaling protein known to prevent cell death in both healthy and cancerous cells. Viral infection triggers innate immunity via proteins known as toll-like receptors (TLR). The researchers showed that TLR activation in mouse immune cells results in a sharp increase in Pim1 expression. Without Pim1, immune cell survival after TLR activation is greatly reduced, highlighting a potentially important role in the antiviral response.

## Introduction

Innate immune cells form the first line of host defense against pathogens because they express molecules called pattern recognition receptors (PRRs) on their surface or endosomes. PPRs recognize viral or bacterial molecules called pathogen-associated molecular patterns (PAMPs). Upon binding of PAMPs, PRRs activate multiple signaling cascades that cause innate immune cells to produce inflammatory cytokines^1–3^ and two key subtypes of the type I interferon (IFN) family, namely, IFN-α and IFN-β; the latter is a highly conserved cytokine that plays critical roles in antiviral innate immune responses^4,5^. Classical examples of PRR-PAMP interactions involve Toll-like receptor 3 (TLR3) and TLR4, which are highly conserved PRRs that recognize double-stranded RNA (dsRNA) and lipopolysaccharide (LPS), respectively^6,7^. These PRR-PAMP interactions induce the selective recruitment of an immune adaptor protein known as toll-interleukin 1 (IL-1) receptor homology (TIR) domain-containing adaptor inducing IFN-β (TRIF), which binds to the TLR and then recruits downstream signaling molecules that ultimately induce the production of IFN-β^8–10^. Specifically, TRIF forms a signaling complex with TNF receptor-associated factor (TRAF) that activates tank-binding kinase 1 (TBK1)^11,12^, which in turn phosphorylates the master transcription factor called interferon regulatory factor 3 (IRF3)^13,14^. This phosphorylation event causes IRF3 to dimerize, translocate into the nucleus, and induce the expression of IFN-β. IFN-β then induces the expression of IFN-stimulated genes (ISGs) through the Janus kinase (JAK)-signal transducer and activator of transcription (STAT) pathway^15,16^.

Pim is a highly conserved serine/threonine kinase with three isoforms, namely, Pim1^17–19^, Pim2^20^ and Pim3^21^. The Pim1 gene contains the upstream CUG start codon, and its Pim1L and Pim1S isoforms are produced by alternative translation^18^. Pim kinases, which have extensive amino acid homology, are constitutively active and play critical roles in multiple cellular functions^22,23^, including cell cycle control^24^, growth^25^, proliferation^26^, migration^27^, apoptosis^28,29^ and survival^30^. Indeed, numerous studies have shown that all Pim kinases are oncogenic proteins that promote tumorigenesis *via* diverse signaling pathways^31–33^. Pim kinases also participate in adaptive immune responses, again *via* disparate mechanisms^34^. For example, Pim1 promotes lymphocyte proliferation and survival by suppressing apoptosis^35^ and promoting NFATc1 activity^36^. Moreover, it enhances CD8 + T-cell survival, promotes CD8 + T-cell memory, and fosters B-cell proliferation^37–39^.

While Pim2 also promotes B-cell survival, it inhibits T-cell immune responses; this is achieved by downregulating T-cell production of pro-inflammatory cytokines^40^. In contrast, while Pim3 also inhibits T-cell responses, it accomplishes this by inhibiting CD4 + and CD8 + T-cell proliferation and activation^41^. In addition, while all Pim kinases are expressed in both Th1 and Th2 cells, their expression is higher in Th1 cells, and they promote Th1-cell differentiation from precursor Th cells^42^.

In contrast, very little is known about the potential role of the Pim kinase family in innate immune responses. Some very limited recent evidence suggests that these kinases can also participate in this arm of the immune system, especially during viral infection. In particular, the results of two studies suggest that Pim1 may be able to modulate virus-induced type I IFN signaling, which is an important mediator of innate immunity^43,44^. Here, we expand this finding by conducting exploratory analyses with Pim1-knockout mice and RNA-seq analyses. We show for the first time that Pim1 facilitates the innate immune responses that are driven by TLR-mediated IFN-β production. Specifically, we observed that Pim1 expression was elevated soon after TLR stimulation and that Pim1 deficiency significantly reduced the phosphorylation and nuclear translocation of IRF3. Our other findings suggest that Pim1 promotes IFN-β expression by enhancing the formation of a cell surface signaling complex composed of TRIF, TRAF3, TBK1, and IRF3; this complex then induces IRF3 phosphorylation. Importantly, the kinase activity of Pim1 was not needed for this function. Our findings thus show that Pim1 can positively regulate the TLR signaling pathway. These observations may provide insights into potential approaches to controlling antiviral responses.

## Materials and Methods

### Mice

*Pim1*^*−/−*^ mice were generated by Macrogen *via* CRISPR/Cas9-mediated genome editing. All mice were on the C57BL/6 genetic background and were bred in the animal facility under specific-pathogen-free conditions. All animal experimental procedures were approved by the Institutional Animal Care and Use Committee (IACUC) of Ewha Womans University (No. 19-006).

### Cells

Murine BMDMs were generated by flushing bone marrow cells from the femurs and tibias of 8- to 10-week-old male C57BL/6 mice, suspending them in Dulbecco’s modified Eagle’s medium (DMEM; HyClone) supplemented with 20% FBS (HyClone), 100 units ml^−1^ penicillin (HyClone) and 100 μg ml^−1^ streptomycin (HyClone), and culturing them at 37 °C and 5% CO_2_ for 1 d. Nonadherent cells were then further cultured with 10 ng ml^−1^ recombinant human macrophage colony-stimulating factor (R&D Systems) for 7 d. *Pim1*^*−/−*^ RAW264.7 cells were generated by ToolGen *via* CRISPR/Cas9-mediated genome editing. A stable HEK293-TLR3 cell line was kindly provided by Dr. I.H. Choi (Yonsei University College of Medicine, Seoul, South Korea). RAW264.7, HEK293-TLR3 and Plat-E cells were cultured in DMEM supplemented with 10% FBS, 100 units ml^−1^ penicillin and 100 μg ml^−1^ streptomycin.

### Reagents and antibodies

BAY 11-7082 (#B5556), BAY 11-7085 (#B5681), PD98059 (#P215), SB203580 (#S8307) and SP600125 (#S5567) were obtained from Sigma‒Aldrich, and SMI-4a (#S8005) was obtained from Selleck Chemicals. Antibodies specific for p-TBK1 (#5483, 1:1,000), TBK1 (#3013, 1:1,000), p-IRF3 (#4947, 1:1,000), IRF3 (#4302, 1:1,000), p-STAT1 (#9167, 1:1,000), p-NF-κB p65 (#3033, 1:1,000), NF-κB p65 (#8242, 1:1,000), p-ERK (#4370, 1:1,000), p-p38 (#9215, 1:1,000) and p-JNK (#9251, 1:1,000) were purchased from Cell Signaling Technology. Antibodies specific for Pim1 (#sc-13513, 1:1,000), Pim2 (#sc-13514, 1:1,000), Pim3 (#sc-98959, 1:1,000), HA (#sc-7392, 1:1,000), GST (#sc-138, 1:1,000), GFP (#sc-9996, 1:1,000), β-actin (#sc-87778, 1:1,000) and GAPDH (#sc-87724, 1:1,000) were obtained from Santa Cruz Biotechnology. A TRIF-specific antibody (#NB120-13810, 1:1000) was purchased from Novus Biologicals, while a Flag-specific antibody (#F3156, 1:10,000) was purchased from Sigma‒Aldrich. Horseradish peroxidase-conjugated secondary antibodies (1:10,000) were obtained from The Jackson Laboratory.

### Cell stimulation

For TLR stimulation, 1 × 10^6^ BMDMs or RAW264.7 cells were seeded in 12-well plates and then treated with 100 ng ml^−1^ Pam3CSK4 (InvivoGen), 10 μg ml^−1^ poly (I:C) (Sigma‒Aldrich) or 100 ng ml^−1^ LPS (Sigma‒Aldrich) for the indicated times.

### Plasmids

The pMX-IRES-EGFP plasmids containing Flag-tagged WT or mutant murine Pim kinase family members and the pIRES-hrGFP-2a plasmid containing HA-tagged murine Pim1 were a gift from Dr. N.S. Kim (Chonnam National University Medical School, Gwangju, Republic of Korea) and have been described previously^45^. The pCMV6 plasmid containing Flag-tagged human TRIF was obtained from Dr. W.S. Ryu (Yonsei University, Seoul, Republic of Korea). The pcDNA3.1 plasmids containing Flag-tagged human WT TBK1 or TBK1 (K38A) and pEGFP-C1 plasmids containing human WT IRF3, IRF3 (5D) or IRF3 (5 A) were gifts from Dr. J.Y. Lee (Gwangju Institute of Science and Technology, Gwangju, Republic of Korea). The pFlag-CMV2 plasmids containing murine TRAF2, TRAF3, TRAF5 or TRAF6 were previously described^46,47^. To generate WT GST-fused human IRF3 and its and deletion mutants, the ORF sequence of IRF3 was amplified from pEGFP-C1-IRF3 by site-directed mutagenesis, and the WT and mutant sequences were subcloned into pEBG plasmids.

### Cell transfection

For plasmid DNA transfection, HEK293-TLR3 or HEK-293T cells were transfected with the indicated plasmids for 36 h by using Lipofectamine 2000 (Invitrogen) according to the manufacturer’s protocol. For siRNA transfection, 1 × 10^6^ BMDMs seeded in 12-well plates were transfected with 20 nM control or murine Pim1-specific siRNA (#AM16708-150114, Ambion) for 36 h by using Lipofectamine RNAiMAX (Invitrogen) according to the manufacturer’s protocol.

### Retroviral transduction

To generate retroviruses, supernatants were collected from Plat-E packaging cells that were transfected with the empty pMX-IRES vector or pMX-IRES-Flag-Pim1 (WT, K67M or DN) plasmid and then filtered through a 0.45-μm filter. For retroviral transduction, 1 × 10^6^ BMDMs seeded in 12-well plates were incubated with retroviral supernatants in the presence of 10 μg ml^−1^ polybrene (Sigma‒Aldrich) for 2 d.

### Immunoprecipitation and immunoblot analysis

Whole-cell lysates were obtained by washing cells with cold PBS (HyClone) and lysing them with RIPA buffer [50 mM Tris-HCl (pH 8.0), 150 mM NaCl, 1% NP-40, 0.1% SDS and 0.5% sodium deoxycholate] containing phosphatase and protease inhibitors. The lysates were immunoprecipitated by incubation with the indicated primary antibodies overnight at 4 °C. Thereafter, they were incubated with protein G Sepharose (Millipore) at 4 °C for 1 h with gentle shaking. The immunoprecipitated proteins were washed with lysis buffer, boiled in 2X SDS sample buffer, separated on 10% SDS-polyacrylamide gels and then electrophoretically transferred onto PVDF membranes (Millipore). The membranes were blocked with 5% BSA and incubated with the indicated primary antibodies and the appropriate HRP-conjugated secondary antibodies.

### GST pulldown assay

HEK293T cells transfected with the indicated combinations of plasmids were washed with cold PBS and lysed with lysis buffer [20 mM HEPES (pH 7.4), 150 mM NaCl, 150 mM KCl, 10 mM EDTA, 10% glycerol and 1% NP-40] containing phosphatase and protease inhibitors. Whole-cell lysates were incubated with glutathione-Sepharose 4B (GE Healthcare) at 4 °C for 1 h with gentle shaking and then washed with lysis buffer. The proteins were boiled in 2× SDS sample buffer and then subjected to immunoblot analysis as described above.

### Cytoplasmic and nuclear fractionation

Cells were lysed with cytoplasmic extraction buffer [10 mM HEPES (pH 7.4), 10 mM KCl, 1.5 mM MgCl_2_, 0.5 M DTT and 0.1% NP-40] containing protease inhibitors. After centrifugation at 8000 rpm for 5 min at 4 °C, the supernatants were collected as the cytoplasmic fraction and boiled in 6× SDS sample buffer. The pellets were lysed on ice for 30 min in nuclear extraction buffer [5 mM HEPES (pH 7.4), 300 mM NaCl, 1.5 mM MgCl_2_, 0.2 mM EDTA and 25% glycerol] containing protease inhibitors. After centrifugation at 14,000 rpm for 30 min at 4 °C, the supernatants were collected as the nuclear fraction and boiled in 6× SDS sample buffer. The cytoplasmic and nuclear fractions were then subjected to immunoblot analysis as described above.

### ELISA

Murine IFN-β levels in cell culture supernatants or murine serum were measured by using mouse IFN beta ELISA kits (Abcam) according to the manufacturer’s instructions.

### Total RNA extraction and RT‒qPCR

Total RNA extracted from cells using the Monarch Total RNA Miniprep Kit (New England Biolabs) was reverse transcribed to cDNA by using PrimeScript RT Master Mix (Takara) according to the manufacturer’s instructions. RT‒qPCR was performed by using the SensiFAST SYBR HI-ROX kit (Bioline) on the Step-One-Plus Real-Time PCR instrument (Applied Biosystems). Target mRNA expression levels were normalized to β-actin mRNA expression levels. Primer sequences are listed in Supplementary Table 1.

### Luciferase assay

HEK293T cells were transiently transfected with the pGL3-IFN-β-luc and pRL-TK *Renilla* luciferase plasmids along with the indicated plasmids. Luciferase activity was measured 24 h post-transfection and normalized to *Renilla* luciferase activity by using the Dual-Luciferase Reporter Assay System (Promega) according to the manufacturer’s instructions.

### Immunofluorescence assay

Cells were fixed with 3.7% paraformaldehyde in PBS for 10 min at room temperature and then permeabilized in 0.2% Triton X-100 in PBS for 10 min at room temperature. After blocking with 1% BSA in PBS for 1 h at room temperature, the cells were stained with an anti-IRF3 antibody (1:500, #NBP2-67741, Novus Biologicals) in PBS overnight at 4 °C and further stained with an Alexa Fluor 488-conjugated goat anti-rabbit antibody (1:1000, Invitrogen) in PBS for 1 h at room temperature. After staining the cells with DAPI for 5 min at room temperature to visualize nuclei, imaging was performed on an ECLIPSE Ts2R fluorescence microscope (Nikon).

### Poly (I:C) injection in mice

WT and *Pim1*^*−/−*^ male mice (8–10 weeks) were injected i.p. with a combination of 2.5 mg kg^−1^ poly (I:C) and 1 g kg^−1^ D-galactosamine diluted in PBS. Serum was obtained for ELISA 6 h post-administration. Survival was monitored every 6 h for 5 d.

### RNA-seq analysis

Total RNA was extracted from WT and *Pim1*^*−/−*^ BMDMs treated with LPS (100 ng ml^−1^) for 4 h with a Monarch Total RNA Miniprep Kit according to the manufacturer’s instructions. Total RNA integrity was measured by using an Agilent Technologies 2100 Bioanalyzer (Agilent) to obtain an RNA integrity number. The mRNA library was prepared with the TruSeq Stranded mRNA Library Prep Kit (Illumina) and sequenced on the NovaSeq 6000 system (Illumina) by DNA Link. To identify differentially expressed genes (DEGs), the raw reads from the RNA-seq library were mapped to the reference genome (Rat rn5) with TopHat (v2.0.13) (http://ccb.jhu.edu/software/tophat), and the aligned results were input into Cuffdiff (v2.2.1) (http://cole-trapnell-lab.github.io/cufflinks/papers). For ontological analysis, genes with a > 2-fold increase in expression, a *p* value of <0.05, and an FDR of <0.1 were selected and subjected to analysis with DAVID (http://david.abcc.ncifcrf.gov). The RNA sequencing data generated during this study are available in GEO (GSE195582) (https://www.ncbi.nlm.nih.gov/geo/query/acc.cgi).

### Statistical analysis

All data are presented as the means ± SDs and represent at least three independent experiments. Groups were compared by two-tailed Student’s *t* test or one-way analysis of variance. GraphPad Prism 8.0 software was used for data analysis.

## Results

### TLR stimuli induce Pim1 expression in macrophages

**Fig. 1:**
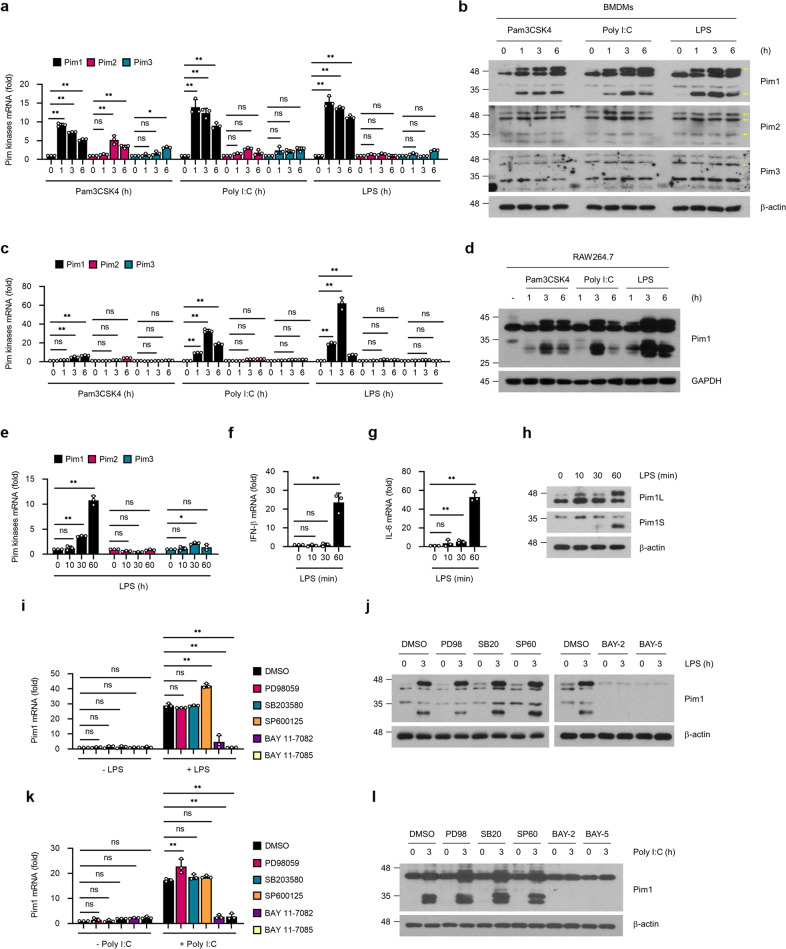
TLR stimulation induces Pim1 expression in macrophages. **a**–**d** Expression of Pim kinases after TLR stimulation. BMDMs **a**, **b**, or RAW264.7 cells **c**, **d** were treated with the TLR2 activator Pam3CSK4 (100 ng ml^−1^), TLR3 activator poly (I:C) (10 μg ml^−1^) or TLR4 activator LPS (100 ng ml^−1^) for the indicated times, and Pim kinase expression was determined by RT‒qPCR **a**, **c** or immunoblot analysis **b**, **d**. The yellow arrows in the immunoblots indicate the Pim isoforms (there are two isoforms, three isoforms, and one isoform of Pim1, Pim2 and Pim3, respectively). **e**–**h** Time course of LPS-induced Pim kinase and TLR4 downstream gene expression. BMDMs were treated with LPS (100 ng ml^−1^) for the indicated times. RT‒qPCR was used to determine Pim kinase **e**, IFN-β **f**, and IL-6 **g** expression. **h** Immunoblot analysis was conducted to determine the expression of the Pim1L and Pim1S isoforms. **i**–**l** Effects of inhibiting specific TLR signaling pathways. BMDMs were preincubated with DMSO, the ERK inhibitor PD98059 (10 μM), the p38 inhibitor SB203580 (10 μM), the JNK inhibitor SP600125 (20 μM), or the NF-κB inhibitor BAY 11-7082 (10 μM) or BAY 11-7085 (10 μM) for 30 min and then treated with LPS (100 ng ml^−1^) **i**, **j** or poly (I:C) (10 μg ml^−1^) **k**, **l** for 3 h. Pim1 expression was determined by RT‒qPCR **i**, **k** or immunoblot analysis **j**, **l**. All mRNA expression values were normalized to β-actin mRNA expression. All data are expressed as the mean ± sd values and are from at least two independent experiments with similar results. *p < 0.05, **p < 0.01 as determined by two-tailed Student’s t test.

To determine whether Pim kinases are involved in innate immune responses, we stimulated TLR2, TLR3 or TLR4 on bone marrow-derived macrophages (BMDMs) or RAW264.7 macrophages with their specific agonists (Pam3CSK4, poly (I:C) and LPS, respectively) and then examined Pim kinase expression. Both quantitative reverse transcription PCR (RT‒qPCR) and immunoblot analysis showed that all three agonists greatly upregulated the mRNA and protein expression of Pim1 but not Pim2 or Pim3 in both macrophage types (Fig. 1a–d).

There are two major adaptors, that are linked to TLR signaling pathways: TRIF and myeloid differentiation factor 88 (MyD88)^1,8,10^. To explore the role of Pim1 in these TLR signaling pathways, we largely focused on the Pim1-TLR4 relationship because TLR4 uses both adaptor molecules. However, we also examined the Pim1-TLR3 relationship in preliminary experiments to explore the role of Pim1 in other IFN I-inducing TLR signaling pathways in macrophages. We did not investigate the Pim1-TLR2 relationship further because TLR2 is not known to be a major type I IFN inducer in macrophages^1,10,48,49^. We first observed that LPS-induced expression of Pim1 started 30 min after LPS stimulation, well before IFN-β or IL-6 expression, which started 1 h after LPS treatment (Fig. 1e–h). It should be noted that LPS induced the expression of both the 44 kDa isoform Pim1L, which is predominantly localized on the cell surface, and the 33 kDa isoform Pim1S, which is mainly localized in the nucleus and cytosol^18,31^

Since the NF-κB and MAPK signaling pathways are also activated soon after TLR signaling^1,5,10^, we then asked whether these pathways drive Pim1 expression. When BMDMs were treated with inhibitors of ERK (PD98059), p38 (SB203580), JNK (SP600125) and NF-κB (BAY 11-7082 and BAY 11-7085), only the NF-κB inhibitors significantly reduced LPS-stimulated Pim1 mRNA and protein expression relative to the DMSO control (Fig. 1I, j). This pattern was also observed for poly (I:C)-stimulated Pim1 expression (Fig. 1k, l). Thus, these TLR stimuli appeared to immediately and specifically induce Pim1 expression in macrophages through the NF-κB pathway.

### Pim1 deficiency reduces TLR-TRIF-mediated IFN-β production

To explore whether Pim1 participates in TLR signaling, we first knocked down Pim1 expression in BMDMs with small interfering RNA (siRNA) (Supplementary Fig. 1a), stimulated the cells with LPS, and used RT‒qPCR to examine the expression of immune response genes that are activated by LPS. Pim1 knockdown significantly decreased the mRNA and protein expression of antiviral response genes, namely, IFN-β, ISG15, ISG54 and ISG56 (Supplementary Fig. 1b), but had no effect on the expression of proinflammatory (TNF-α, IL-1β, and IL-6) or anti-inflammatory (IL-10) cytokines (Supplementary Fig. 1c). A similar analysis of CRISPR‒Cas9-generated *Pim1*^*−/−*^ RAW264.7 cell clones confirmed that Pim1 regulates LPS-stimulated IFN-β and ISG mRNA expression (Supplementary Fig. 2a, b). This effect was also observed when the stimulant was poly (I:C) (Supplementary Fig. 2c, d). Thus, Pim1 may play an important role in TLR4- and TLR3-mediated antiviral gene expression.

**Fig. 2:**
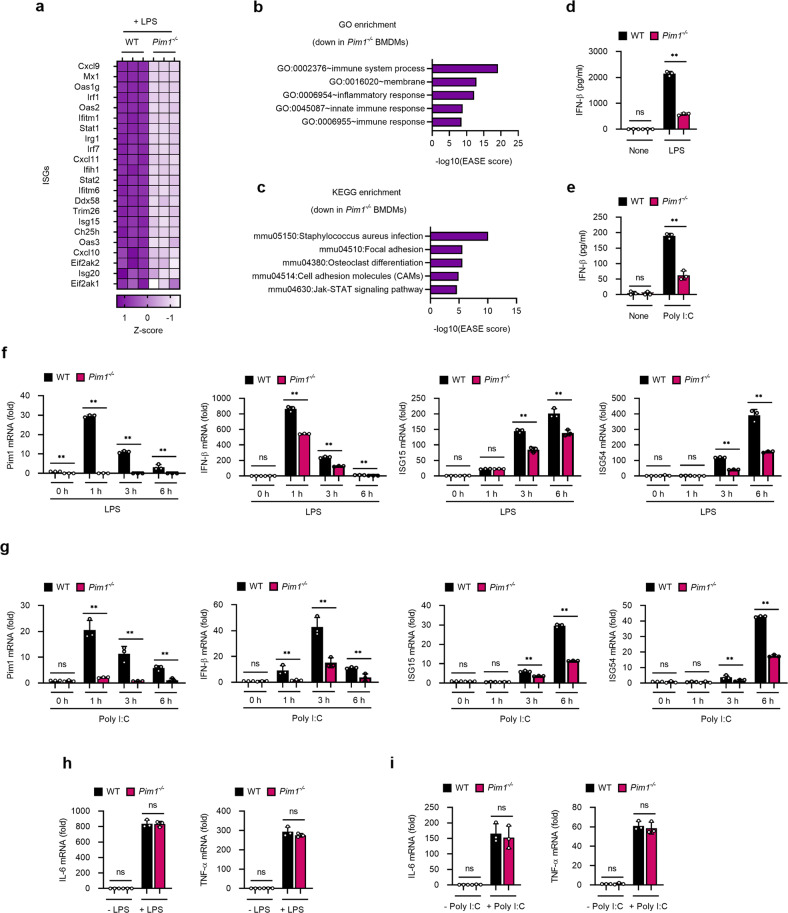
Pim1 deficiency in vivo reduces TLR-mediated antiviral gene expression. Effect of in vivo Pim1 deletion on the RNA profiles **a**–**c** and IFN-β and ISG expression **d**–**i** in TLR-stimulated BMDMs. **a**–**c** Three WT and three *Pim1*^*−/−*^ BMDM replicates were treated with LPS (100 ng ml^−1^) for 4 h. **a** Heatmap of the downregulated ISGs in *Pim1*^*−/−*^ BMDMs. The selected ISGs are ordered according to their Z score. **b** GO term enrichment analysis. **c** KEGG pathway enrichment analysis. The top five pathways are ordered according to their EASE score. **d**, **e** ELISA-determined IFN-β levels in the supernatants of WT and *Pim1*^*−/−*^ BMDMs treated with LPS (100 ng ml^−1^) **d** or poly (I:C) (10 μg ml^−1^) **e** for 6 h. **f**–**i** RT‒qPCR of Pim1, IFN-β, ISG15, ISG54 **f**, **g**, IL-6 and TNF-α **h**, **i** in WT and *Pim1*^*−/−*^ BMDMs treated with LPS (100 ng ml^−1^) **f**, **h** or poly (I:C) (10 μg ml^−1^) **g**, **i** for the indicated times. mRNA expression values were normalized to β-actin mRNA expression. All data are presented as the mean ± sd values and are from at least two independent experiments with similar results. **p < 0.01 as determined by two-tailed Student’s *t* test.

To determine whether Pim1 plays a similar role in innate immune responses in vivo, CRISPR‒Cas9 genome editing was used to generate *Pim1*^*−/−*^ mice, which have a large deletion (exons I–V) in the *Pim1* gene. The *Pim1*^*−/−*^ mice were viable, fertile and had normal teeth (data not shown). Flow cytometric analyses indicated that the neutrophil, dendritic cell, and macrophage frequencies in the bone marrow of *Pim1*^*−/−*^ mice were comparable to those in wild-type mice, suggesting that Pim1 deletion did not affect the development of innate immune cells (Supplementary Fig. 3). To determine whether in vivo Pim1 deletion also impairs antiviral gene expression in macrophages, we isolated BMDMs from WT and *Pim1*^*−/−*^ mice, treated them with LPS, and then performed RNA-seq analysis. Indeed, differential gene expression analysis showed that Pim1 deletion significantly reduced ISG expression (Fig. 2a), while GO and KEGG enrichment analyses showed that the top five terms enriched with genes downregulated in *Pim1*^*−/−*^ BMDMs were related to innate immune responses (Fig. 2b, c). Moreover, when *Pim1*^*−/−*^ BMDMs were subjected to TLR stimulation (LPS or poly (I:C)), ELISA showed that *Pim1*^*−/−*^ BMDMs produced significantly less IFN-β than similarly treated WT BMDMs regardless of the TLR agonist (Fig. 2d, e). Similarly, *Pim1*^*−/−*^ BMDMs exhibited drastically reduced mRNA levels of Pim1, IFN-β, ISG15 and ISG56 (Fig. 2f, g) but not of IL-6 and TNF-α (Fig. 2h, i) after LPS or poly (I:C) stimulation when compared to WT cells. It should be noted that deletion of Pim1 did not completely abolish LPS- or poly (I:C)-induced ISG signaling and IFN-β production (Fig. 2d–g). This was also observed when Pim1 was knocked down in BMDMs (Supplementary Fig. 1b). Thus, Pim1 appears to augment TLR4- and TLR3-mediated gene expression rather than be essential for its initiation.

Virus-induced IFN-β expression is mediated not only by TLRs but also by two other PPRs, namely, the cytosolic molecules retinoic acid-inducible gene 1 (RIG-I)-like receptor (RLR) and cyclic GMP-AMP synthase (cGAS)^4,9^. RLR recognizes cytosolic PAMP RNA and induces IRF3 phosphorylation by modulating the adaptor protein mitochondrial antiviral signaling protein (MAVS)^50^; cGAS recognizes cytosolic PAMP DNA and induces IRF3 phosphorylation by modulating stimulator of interferon genes (STING)^51^. Recently, Zhang et al. showed that Pim1 downregulates Sendai virus (SeV)-induced IFN-β activation by inhibiting RIG-I-mediated signaling^43^. This, together with the observation that the TLR-TRIF, RLR-MAVS, and cGAS-STING-mediated signaling pathways share some components, led us to examine the role of Pim1 in RLR and cGAS signaling in BMDMs. Similar to treatment with TLR2, TLR3, and TLR4 agonists (Fig. 1a), treatment with agonists specific for either RLR [poly (I:C) transfection] or cGAS (2′3′-cGAMP transfection) stimulated Pim1 mRNA expression in WT BMDMs (Supplementary Fig. 4a, b, left-hand panels). Moreover, as expected, both stimuli upregulated IFN-β protein production and mRNA expression and IL-6 and TNF-α mRNA expression in WT BMDMs (Supplementary Fig. 4a–d). However, Pim1 deletion showed that Pim1 acted quite differently in RLR and cGAS signaling: Pim1^*−/−*^, associated with a further increase in RLR-mediated IFN-β, IL-6, and TNF-α production, had no effect on cGAS-mediated IFN-β production but downregulated cGAS-mediated IL-6 and TNF-α expression (Supplementary Fig. 4a–d). Thus, while Pim1 is upregulated in macrophages by specific agonists of TLR, RLR, and cGAS signaling, it plays quite different roles in the inflammatory outcomes of these pathways: (i) in the TLR pathway, it promotes IFN-β expression but has no effect on IL-6 and TNF-α expression; (ii) in the RLR pathway, it inhibits IFN-β, IL-6 and TNF-α expression; and (iii) in the cGAS pathway, it has no effect on IFN-β expression but increases IL-6 and TNF-α expression. These results suggest that Pim1 may shape the specificity of TLR- and RLR-mediated IFN-β production.

### Pim1 promotes TLR4-mediated IFN-β production via the TRIF-TBK1-IRF3 axis in a kinase activity-independent manner

**Fig. 3:**
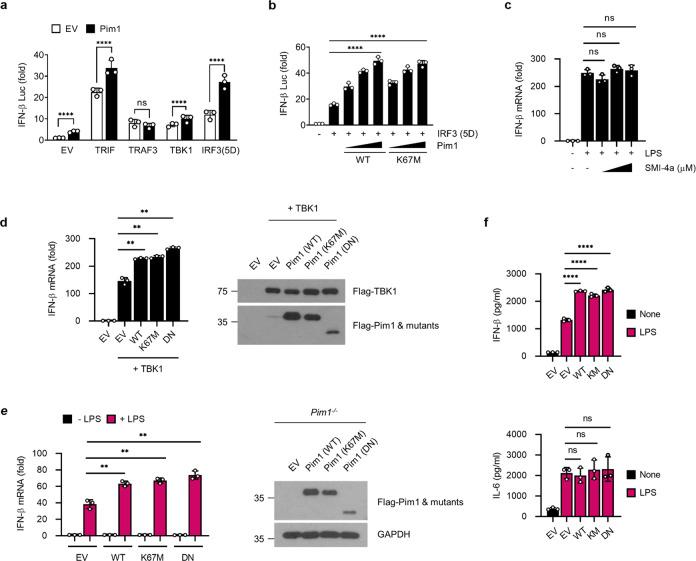
Pim1 positively regulates TLR4-mediated IFN-β production in a kinase activity-independent manner. **a** Ability of Pim1 to enhance the IFN-β expression induced by TLR downstream signaling molecules or constitutively active IRF3 (5D). HEK293T cells were transfected for 24 h with an IFN-β promoter-driven luciferase reporter, internal control *Renilla* luciferase reporter, and Flag-Pim1 plasmid [or its empty vector (EV)] together with plasmids expressing Flag-TRIF, Flag-TRAF3, Flag-TBK1, EGFP-IRF3 (5D) or the corresponding EVs. Luciferase values are presented as fold induction relative to the values in EV-transfected cells. **b**–**f** Role of the kinase activity of Pim1 in its ability to promote IFN-β expression. In **b**, HEK293T cells were transfected with the IFN-β luciferase reporter and EGFP-IRF3 (5D) as described in **a** along with increasing amounts of the Flag-Pim1 WT or K67M plasmid. The Pim1 K67M mutant lacks kinase activity. Luciferase activity was measured as described in **a**. In **c**, BMDMs were preincubated with increasing amounts of the Pim1 kinase inhibitor SMI-4a, treated with LPS (100 ng ml^−1^) for 3 h, and then subjected to RT‒qPCR analysis of IFN-β. In **d**, HEK293T cells were transfected with Flag-Pim1 or its K67M or double-negative (DN) mutant along with Flag-TBK1 and were then subjected to RT‒qPCR analysis of IFN-β (left) or immunoblot analysis of the plasmid constructs (right). In **e**, **f**, *Pim1*^*−/−*^ BMDMs were transduced with retroviral vectors expressing Flag-tagged WT or mutant Pim1 and treated with LPS (100 ng ml^−1^) for 3 h or 6 h. After 3 h, the cells were subjected to RT‒qPCR analysis of IFN-β (**e**, left) or immunoblot analysis of Pim1 (**e**, right). After 6 h, the IFN-β and IL-6 levels in the supernatant were measured with ELISA **f**. All mRNA expression values were normalized to β-actin mRNA expression. All data are presented as the mean ± sd values and are from at least two independent experiments with similar results. **p < 0.01, ****p < 0.0001 as determined by two-tailed Student’s *t* test.

LPS induces IFN-β expression via the TLR4-TRIF pathway: specifically, LPS-bound TLR4 recruits TRIF, which forms a complex with TRAF that activates TBK1, which in turn phosphorylates IRF3 and induces its nuclear translocation and IFN-β transcription^9–11^. To identify the mechanism by which Pim1 regulates IFN-β production, HEK293T cells were transfected with an IFN-β promoter-driven luciferase reporter, the internal control *Renilla* luciferase reporter, and Flag-tagged Pim1 along with plasmids expressing the upstream kinases that are involved in TLR4-mediated signaling (TRIF, TRAF3 and TBK1). Alternatively, the cells were cotransfected with a plasmid expressing constitutively active IRF3 (5D)^52^. Compared to the empty vector, the Pim1 expression plasmid significantly increased TRIF-, TBK1-, and 5D-induced IFN-β-driven luciferase activity but not TRAF3-induced IFN-β-driven luciferase activity (Fig. 3a). This suggests that Pim1 regulates IFN-β production through the TRIF-TBK1-IRF3 axis.

To test whether the kinase activity of Pim1 is required for its ability to regulate IRF3, we transfected luciferase reporter-expressing HEK293T cells with IRF3 5D and a kinase-dead Pim1 mutant (K67M)^45^ instead of WT Pim1. The mutant induced the same level of IFN-β-luciferase activity as WT Pim1 (Fig. 3b). Similarly, coculturing BMDMs with SMI-4a, which inhibits Pim1 kinase activity^23^, did not affect their LPS-induced IFN-β mRNA levels (Fig. 3c). Moreover, the ability of WT Pim1 to enhance the ability of the TBK1 expression plasmid to induce IFN-β mRNA expression in HEK293T cells was reproduced when WT Pim1 was replaced with the kinase-dead (K67M) or dominant-negative (DN) mutant of Pim1 (Fig. 3d). In addition, *Pim1*^*−/−*^ BMDMs that were transduced with retroviral vectors encoding WT, K67M or DN Pim1 produced the same amounts of IFN-β mRNA and protein after LPS treatment (Fig. 3e). In contrast, neither WT Pim1 nor K67M or DN Pim1 enhanced the production of IL-6 in *Pim1*^*−/−*^ BMDMs after LPS stimulation (Fig. 3f). Thus, the kinase activity of Pim1 does not appear to be needed for its role in TLR4-mediated IFN-β production.

### Pim1 positively regulates IRF3 phosphorylation and nuclear translocation

**Fig. 4:**
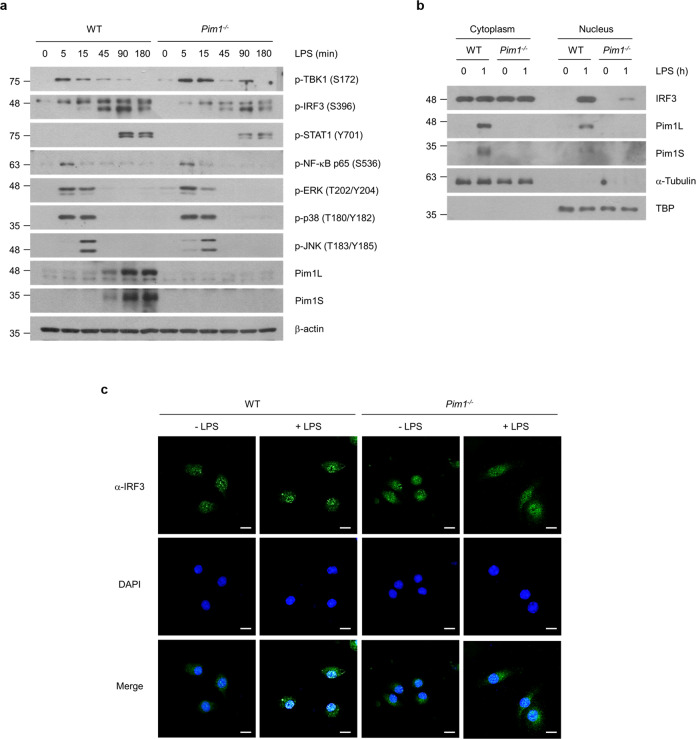
Pim1 promotes the phosphorylation and nuclear translocation of IRF3. **a** Ability of Pim1 to affect LPS-induced phosphorylation of TLR4 upstream kinases or downstream IRF3. WT or *Pim1*^*−/−*^ BMDMs were treated with LPS (100 ng ml^−1^) for the indicated times and were then subjected to immunoblot analysis of phosphorylated TBK1, IRF3, STAT1, NF-κB p65, ERK, p38 and JNK. **b**, **c** Ability of Pim1 to promote the nuclear translocation of IRF3. WT and *Pim1*^*−/−*^ BMDMs were treated with LPS (100 ng ml^−1^) for 1 h. The cytoplasmic and nuclear fractions of these cells were isolated and subjected to immunoblot analysis **b**, or the intact cells were used for immunofluorescence analysis of endogenous IRF3 with DAPI counterstaining of nuclei. Scale bars, 10 μm. All data are from at least two independent experiments with similar results.

To determine how Pim1 regulates TLR4-mediated IFN-β expression, we asked whether its deletion affects the phosphorylation of the protein kinases that are involved in TLR4-mediated signaling. Notably, *Pim1*^*−/−*^ BMDMs exhibited decreased IRF3 and STAT1 phosphorylation after LPS treatment compared with WT BMDMs, but phosphorylation of the kinases (TBK1, NF-κB, ERK, p38, and JNK) was unaffected (Fig. 4a). Since STAT1 acts downstream and the kinases act upstream of IRF3^15,16^, it appears that Pim1 regulates IRF3 rather than its kinases. This hypothesis was supported by immunoblot analysis of the cytosolic and nuclear fractions of *Pim1*^*−/−*^ and WT BMDMs: deletion of Pim1 significantly reduced the LPS-induced nuclear translocation of IRF3 (Fig. 4b). *Pim1*^*−/−*^ BMDMs also exhibited noticeably less nuclear IRF3 immunofluorescence after LPS treatment than WT BMDMs (Fig. 4c). In contrast, WT and *Pim1*^*−/−*^ BMDMs displayed similar TBK1 and NF-κB p65 immunofluorescence patterns (Supplementary Fig. 5a, b). Thus, Pim1 appears to positively and directly regulate IRF3 phosphorylation in a manner that is independent of its kinase activity.

### Pim1 associates with TRIF, TRAF3, TBK1, and IRF3

**Fig. 5:**
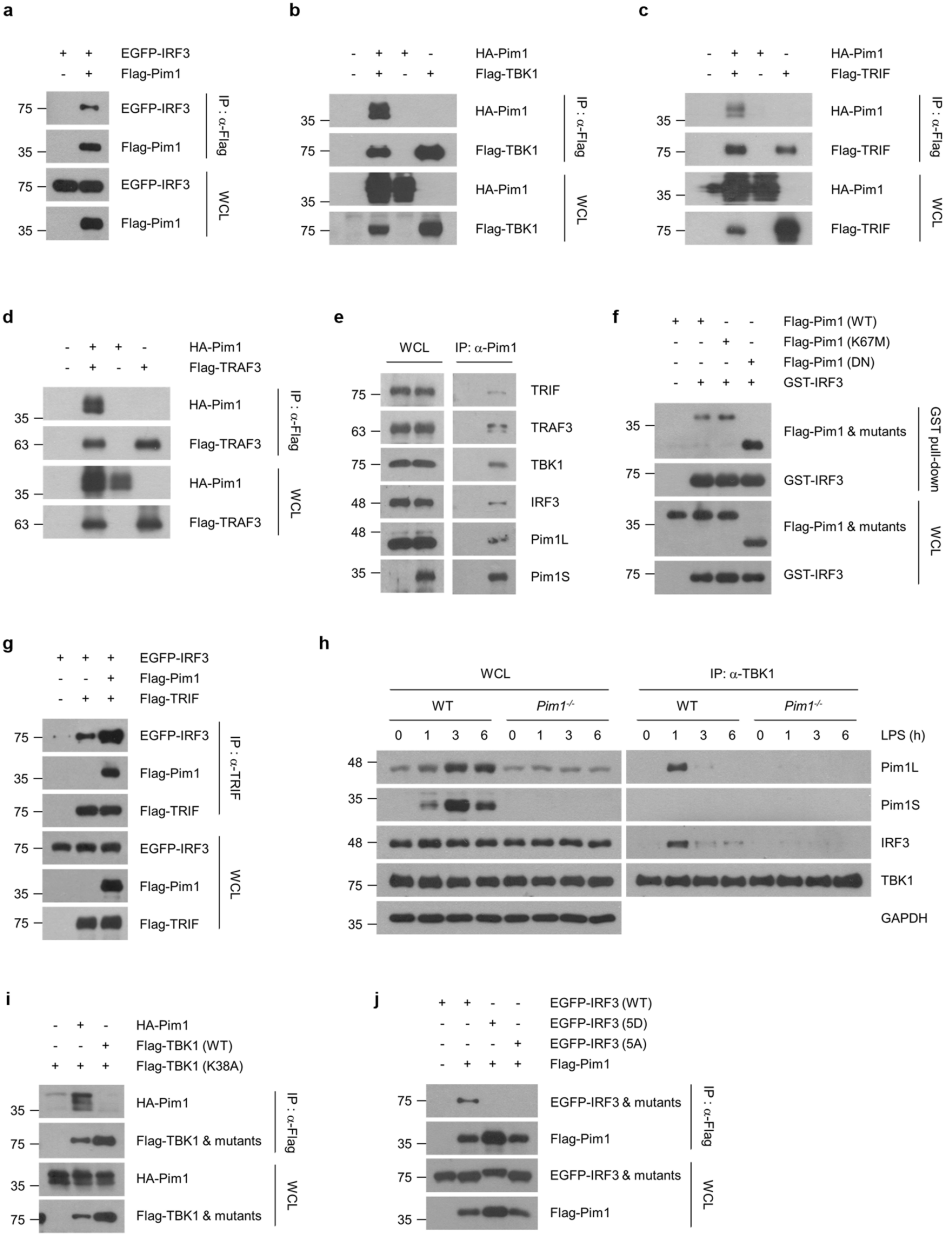
Pim1 promotes the formation of the TRIF-TRAF3-TBK1-IRF3 signaling complex. **a**–**d** Ability of Pim1 to interact with IRF3 and its upstream activators. HEK293T cells were transfected with differently tagged Pim1 constructs plus IRF3 **a**, TBK1 **b**, TRIF **c**, or TRAF3 **d**, and were then incubated with the indicated tag-specific antibody. The immunoprecipitates were then subjected to immunoblot analysis. **e** Pim1 interacts endogenously with the TRIF-TRAF3-TBK1-IRF3 signaling complex. BMDMs were stimulated with LPS (100 ng ml^−1^) for 1 h and subjected to immunoprecipitation with an anti-Pim1 antibody. TRIF, TRAF3, TBK1, IRF3 and Pim1 levels were determined by immunoblotting. **f** Role of Pim1 kinase activity in the interaction between Pim1 and IRF3. HEK293T cells were transfected with GST-tagged IRF3 and Flag-tagged Pim1 WT or mutant plasmids and subjected to a GST pulldown assay. **g** Ability of Pim1 to form a ternary complex with TRIF and IRF3. HEK293T cells were transfected with differently tagged Pim1, TRIF and IRF3 constructs and were then subjected to an immunoprecipitation assay. **h** Ability of Pim1 to form a ternary complex with TBK1 and IRF3. WT and *Pim1*^*−/−*^ RAW264.7 cells were treated with LPS (100 ng ml^−1^) for the indicated times, after which immunoprecipitation analysis was conducted with an antibody against endogenous TBK1. **i**, **j** Ability of Pim1 to interact with the kinase-inactive TBK1 (K38A) mutant **i** and the phosphomimetic (5D) and phosphorylation-defective (5 A) mutants of IRF3 **j**. HEK293T cells were transfected with HA-Pim1 plus WT or kinase-inactive TBK1 **i** or WT, 5D or 5 A IRF3 **j** and were then subjected to an immunoprecipitation assay with the indicated antibody. All data are from at least two independent experiments with similar results. *IP* immunoprecipitation, *WCL* whole-cell lysate.

To determine precisely how Pim1 regulates IRF3 phosphorylation, we conducted a series of immunoprecipitation and GST pulldown assays to assess the ability of Pim1 to interact with IRF3 and its upstream activators TRIF, TBK1, and TRAF3^53,54^. First, HEK293T cells were transfected with differently tagged Pim1 and IRF3, TRIF, TBK1 or TRAF3 and then subjected to immunoprecipitation assays using tag-specific antibodies. Pim1 coimmunoprecipitated with IRF3, TBK1, TRIF, and TRAF3 (Fig. 5a–d). Second, coimmunoprecipitation experiments were conducted with LPS-stimulated WT BMDMs and an anti-Pim1 antibody to determine whether Pim1 associates with endogenous TRIF, TRAF3, TBK1 or IRF3. Indeed, Pim1 associated with TRIF, TRAF3, TBK1, and IRF3 under these physiological conditions (Fig. 5e).

Since the kinase activity of Pim1 was not necessary for its induction of IFN-β expression (Fig. 3b–f), we hypothesized that Pim1 kinase activity is not involved in its interaction with IRF3. Indeed, GST pulldown assays with HEK293T cells that were transfected with GST-tagged IRF3 and WT or mutant Pim1 showed that both kinase-dead and dominant-negative Pim1 were pulled down with IRF3 (Fig. 5f). Since IRF3 binds to TRIF after LPS stimulation^9^, we next asked whether Pim1 promotes TRIF-IRF3 complex formation. Indeed, immunoprecipitation assays in HEK293T cells showed that TRIF and IRF3 interact, that Pim1 forms a ternary complex with TRIF and IRF3, and that Pim1 significantly enhances the TRIF-IRF3 interaction (Fig. 5g).

After IRF3 is recruited to TRIF, TBK1 phosphorylates IRF3^9^. To determine whether Pim1 participates in the LPS-induced formation of the TBK1-IRF3 signaling complex, *Pim1*^*−/−*^ and WT RAW264.7 cells were treated with LPS and then subjected to immunoprecipitation with an antibody that recognizes endogenous TBK1. The results showed that soon after LPS treatment (1 h), a ternary complex containing TBK1, IRF3, and Pim1L formed. Moreover, Pim1 deletion markedly abrogated the interaction between TBK1 and IRF3 (Fig. 5h). Since Pim1L is primarily localized on the plasma membrane and Pim1S localizes to both the cytoplasm and nucleus^18,31^, our finding suggests that Pim1 quickly complexes with TBK1 and IRF3 on the plasma membrane after LPS treatment. We also observed by an immunoprecipitation assay that Pim1 did not interact with kinase-inactive TBK1 (K38A) (Fig. 5i) or with either the phosphomimetic (5D) or phosphorylation-defective (5 A) mutant of IRF3 (Fig. 5j). The latter observations are consistent with the observation that phosphorylation of IRF3 causes it to dissociate from its signaling complex and translocate into the nucleus^9,55^. Thus, Pim1 appears to form a signaling complex containing TRIF, TRAF3, TBK1, and IRF3 at the cell surface.

### Pim1-deficient mice are more susceptible to poly (I:C)-induced death

**Fig. 6:**
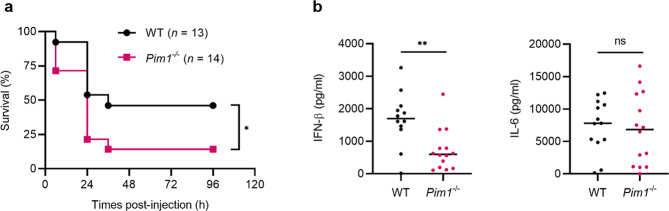
Pim1-deficient mice display greater susceptibility to poly (I:C)-induced death. WT and *Pim1*^*−/−*^ mice were challenged with poly (I:C) (2.5 mg kg^−1^) plus _D_-galactosamine (1 g kg^−1^). **a** Animal survival was monitored every 6 h for 5 d (WT, *n* = 13; *Pim1*^*−/*^^−^, *n* = 14). **b**, IFN-β and IL-6 levels in the serum of the mice 6 h after injection (WT, *n* = 11; *Pim1*^*−/−*^, *n* = 13). The data are presented as the mean ± sd values and are from at least two independent experiments with similar results. *p < 0.05 as determined by using the log-rank (Mantel‒Cox) test; **p < 0.01 as determined by two-tailed Student’s t test.

Since Pim1-deficient BMDMs stimulated with TLR3 or TLR4 produced little IFN-β but had normal inflammatory cytokine levels (Fig. 2f–i), we asked whether Pim1 regulates innate immune responses in vivo. To this end, we injected WT and *Pim1*^*−/−*^ mice intraperitoneally with poly (I:C) and _D_-galactosamine and then monitored the resulting levels of TLR3-induced inflammatory cytokines in the serum and the survival of the mice. Indeed, *Pim1*^*−/−*^ mice had lower serum IFN-β levels but normal serum IL-6 levels and a significantly lower survival rate (Fig. 6). This is consistent with the in vitro observations above, namely, that Pim1 is needed for TLR-mediated IFN-β production but not IL-6 production. Since poly (I:C) is a well-known mimic of pathogenic TLR-activating viruses and IFN-β is a key antiviral cytokine, these findings suggest that Pim1 can regulate the antiviral response in vivo.

## Discussion

Our in vitro and in vivo experiments suggest that Pim1, which is well known for its oncogenic functions^23,31–33^, is also a novel regulator of an important innate immune antiviral response, namely, TLR-induced ISG expression and IFN-β production. Specifically, we found that Pim1 was quickly upregulated by TLR-mediated signaling in an NF-κB-dependent manner and that Pim1 expression was associated with IFN-β-mediated immune responses but not pro- or anti-inflammatory cytokine responses. We then showed that Pim1 promoted IFN-β production by positively regulating the phosphorylation and nuclear translocation of IRF3. However, it did not do so by phosphorylating IRF3 or by phosphorylating the IRF3 kinase TBK1. It also did not affect the IRF3 upstream adaptor TRAF3, since Pim1 expression did not change the ability of TRAF3 to activate the IFN-β promoter. Rather, Pim1 seemed to act by enhancing the formation of complexes between IRF3, its upstream adaptors TRIF and TRAF3, and its kinase TBK1. Furthermore, the in vitro findings were reproduced in vivo: when *Pim1*^*−/−*^ mice were subjected to poly (I:C)-induced lethality, they produced less serum IFN-β (but not less IL-6) and demonstrated poorer survival than WT mice.

A handful of recent studies support the idea that Pim1 can shape innate immune responses to viruses^43,44,56–60^, although the mechanisms and outcomes vary widely. Specifically, two studies showed that Pim1 inhibits virus propagation by increasing the population of neutrophils that diminish CD8 + T-cell-mediated suppression of viruses^59^ or by increasing host cell death^56^. Conversely, four other studies reported that Pim1 promotes virus propagation by facilitating viral entry^57^ or viral translation^58^ or, interestingly, by decreasing the IFN-β signaling in host or immune cells (CD4 + T cells, monocytes, and B cells) that is induced by Zika virus^44^ or Sendai virus^43^. However, it should be noted that all of these virus-modulating effects of Pim1, including IFN-β downregulation, are mediated by its kinase activity; in contrast, our study showed that the ability of Pim1 to upregulate TLR-induced IFN-β production was independent of its kinase activity.

The observation that Pim1 can both upregulate and downregulate IFN-β production may reflect the ability of Pim1 to shape different IFN-β-stimulating pathways. Thus, while we showed that Pim1 enhances TLR-mediated IFN-β production by complexing with IRF3 and its adaptors and kinase, the results of the Zika and Sendai virus studies suggested that Pim1 inhibits virus-mediated IFN-β promoter activation by phosphorylating a molecule upstream of TBK1 and thereby blocking the RIG-I pathway (however, the RIG-I protein level was not affected)^43,44^. This is supported by our preliminary analyses of RLR signaling, which indicated that Pim1 downregulated RLR-mediated IFN-β production. Interestingly, our preliminary analyses also showed that Pim1 had no effect on cGAS-mediated IFN-β production. This variable role of Pim1 in IFN-β production was also mirrored by its disparate contributions to proinflammatory cytokine expression after activation of TLRs (no effect), RLR (downregulation), and cGAS (upregulation). Notably, this variable role of Pim1 is reminiscent of the diverse mechanisms by which Pim kinases promote tumorigenesis^31–33^. Thus, although our RLR or cGAS findings remain to be confirmed and extended with analyses of kinase-deficient Pim1, they tentatively suggest that targeting the kinase-independent interaction between Pim1 and the IRF3 signaling complex could help control viral infections. These findings also confirm that although the TLR, RLR, and cGAS pathways regulate IFN-β production via common players, they are tightly and selectively controlled, thereby inducing appropriate antiviral responses. Our study thus suggests that Pim1 may act as a specificity guide in these pathways.

This idea was further supported by our finding that Pim1 can interact with multiple members of the TRAF family, including TRAF2, TRAF5, and TRAF6 (Supplementary Fig. 6a, b). TRAFs are recruited by PPR-activated TRIF, after which they phosphorylate TBK1, which in turn phosphorylates the TRIF in the complex and causes IRF3 to bind to this TRIF^9,46^. Several studies suggest that TRAF family members contribute to the specificity of the immune response. For example, TRAF2, TRAF5 and TRAF6 are required for TRIF-mediated or MAVS-mediated but not STING-mediated TBK1 activation^9^^,^ whereas TRAF3 regulates TLR-mediated and RLR-mediated type I IFN responses^53,54^. The possibility that TRAF3 acts with Pim1 to endow the IRF3 signaling complex with selectivity is further supported by our finding that although Pim1 interacts with TRAF3, it does not increase the ability of TRAF3 to activate the IFN-β promoter in HEK293T cells.

Our finding that Pim1 promotes IRF3 phosphorylation by promoting the association between IRF3 and its upstream kinases rather than by phosphorylating IRF3 is reminiscent of other mechanisms that regulate IFN-β production^11,12^. For example, GSK3β positively regulates virus-induced IRF3 activation and IFN-β production by promoting the association of TBK1 with IRF3 or the TRAF2-mediated ubiquitination of IRF3 in a kinase-independent manner^46,47,61^. Notably, our study also showed that Pim1 interacts with TBK1, but Pim1 deletion did not affect the LPS-induced phospho-TBK1 level. This is consistent with the idea that in this setting, Pim1 acts to bring together various players in IFN-β signaling, thereby promoting the selectivity of the signaling complex.

Finally, our study showed that while Pim1 expression enhanced the interaction between TBK1 and IRF3, only the Pim1L isoform complexed with TBK1 and IRF3, although both isoforms were expressed after TLR stimulation. Since Pim1L but not Pim1S contains a proline-rich motif at its N-terminus^18,31^, this motif may play a key role in the interaction between TBK1 and IRF3. These data indicate that Pim1 isoforms may play different roles in antiviral responses.

In conclusion, this study showed that Pim1 plays a key role in TLR-mediated IFN-β and ISG production. Specifically, after TLR stimulation, Pim1 is upregulated and promotes IFN-β production by promoting the association between IRF3 and its upstream adaptors and kinases. In summary, our study indicates that Pim1 may serve as a crucial positive regulator of innate immune antiviral responses.

## Supplementary information

Supplemental Information
