# Supplementary material for: Pim1 promotes IFN-β production by interacting with IRF3
Source: Exp Mol Med. 2022 Nov 29;54(11):2092–103. doi: 10.1038/s12276-022-00893-y (PMC9722908; doi:10.1038/s12276-022-00893-y)
Supplement: Supplementary file 1 — Supplemental Information [file 12276_2022_893_MOESM1_ESM.pdf]

Supplemental Information

Pim1 promotes IFN-β production by interacting with IRF3

(Running title: Positive regulation of IFN-β production by Pim1)

Ryeojin Ko[1,2], Jeongin Seo[1], Hana Park[1], Nawon Lee[1] and Soo Young Lee[1,2,*]

[1]Department of Life Science, Ewha Womans University, Seoul 03760, Korea.

[2]The Research Center for Cellular Homeostasis, Ewha Womans University, Seoul 03760, Korea.

[*]Correspondence should be addressed to:

SY Lee, Department of Life Science and the Research Center for Cellular Homeostasis, Ewha Womans University, Seoul 03760, South Korea. Tel: 82-2-3277-3770; Fax: 82-2-3277-3760; E-mail: leesy@ewha.ac.kr

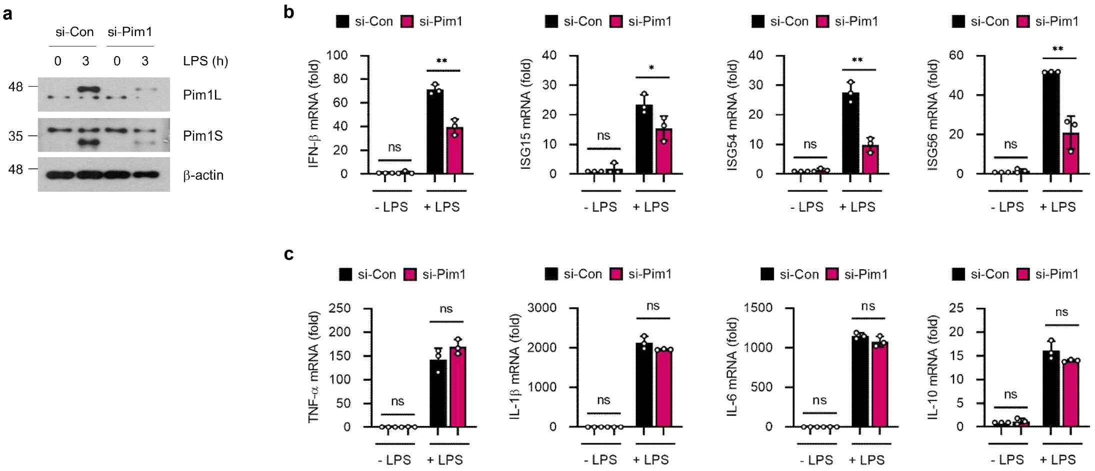

Supplementary Fig. 1. Pim1 positively regulates TLR-mediated antiviral gene expression.

a-c, Effect of Pim1 silencing. BMDMs were transfected with control siRNA (si-Con) or Pim1-specific siRNA (si-Pim1) and treated with LPS (100 ng ml^{-1}) for 3 h, after which Pim1 (**a**), IFN-β, ISG15, ISG54, ISG56 (**b**), or TNF-α, IL-1β, IL-6 and IL-10 (**c**) expression was determined by immunoblot analysis (**a**) or RT-qPCR (**b**,**c**). All mRNA values were normalized to β-actin mRNA expression. All data are expressed as mean ± s.d. and represent at least three independent experiments with similar results. *$p < 0.05$, **$p < 0.01$, as determined by two-tailed Student's t-test.

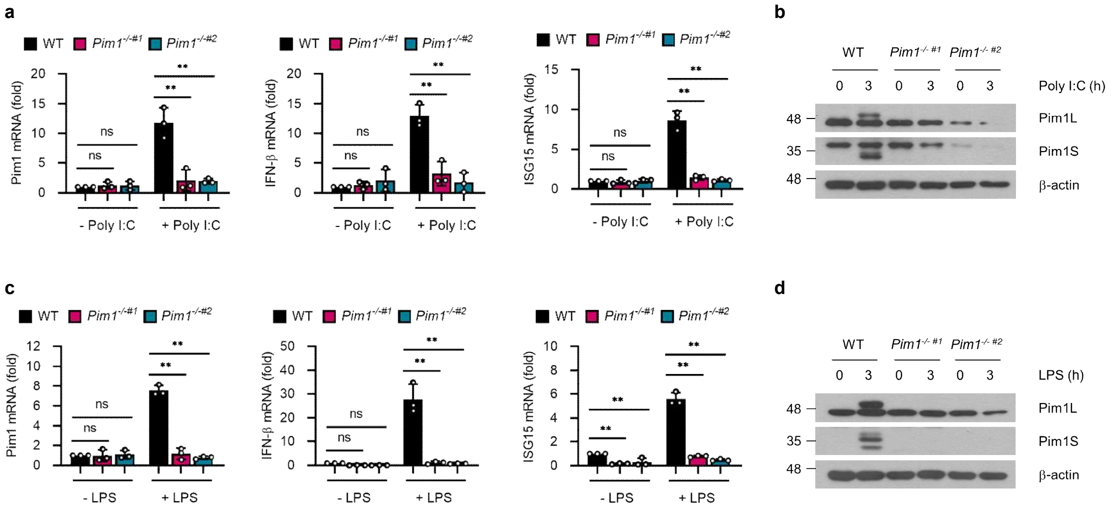

Supplementary Fig. 2. Pim1 deficiency reduces TLR-mediated antiviral gene expression in RAW264.7 cells.

Two *Pim1*$^{-/-}$ RAW264.7 cell clones were generated. Both *Pim1*$^{-/-}$ clones and WT RAW264.7 cells were subjected to TLR stimulation with LPS (**a,b**) or poly I:C (**c,d**) for the indicated times, after which Pim1, IFN-β or ISG15 RT-qPCR (**a,c**) or Pim1 immunoblot (**b,d**) was conducted. All mRNA values were normalized to β-actin mRNA expression. All data are presented as mean ± s.d. and represent at least three independent experiments with similar results. **p* < 0.05, ***p* < 0.01, as determined by two-tailed Student's t-test.

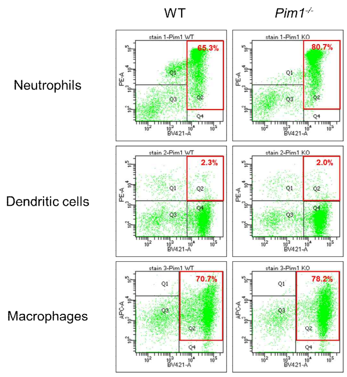

Supplementary Fig. 3. Characterization of *Pim1*$^{-/-}$ mice.

Flow cytometric analysis of bone marrow cells from WT and *Pim1*$^{-/-}$ mice at 8 weeks of age to determine neutrophil (CD11b/Gr1+), dendritic cell (CD11b/CD11c+) and macrophage (CD11b/F4/80+) frequencies. All data represent at least two independent experiments with similar results.

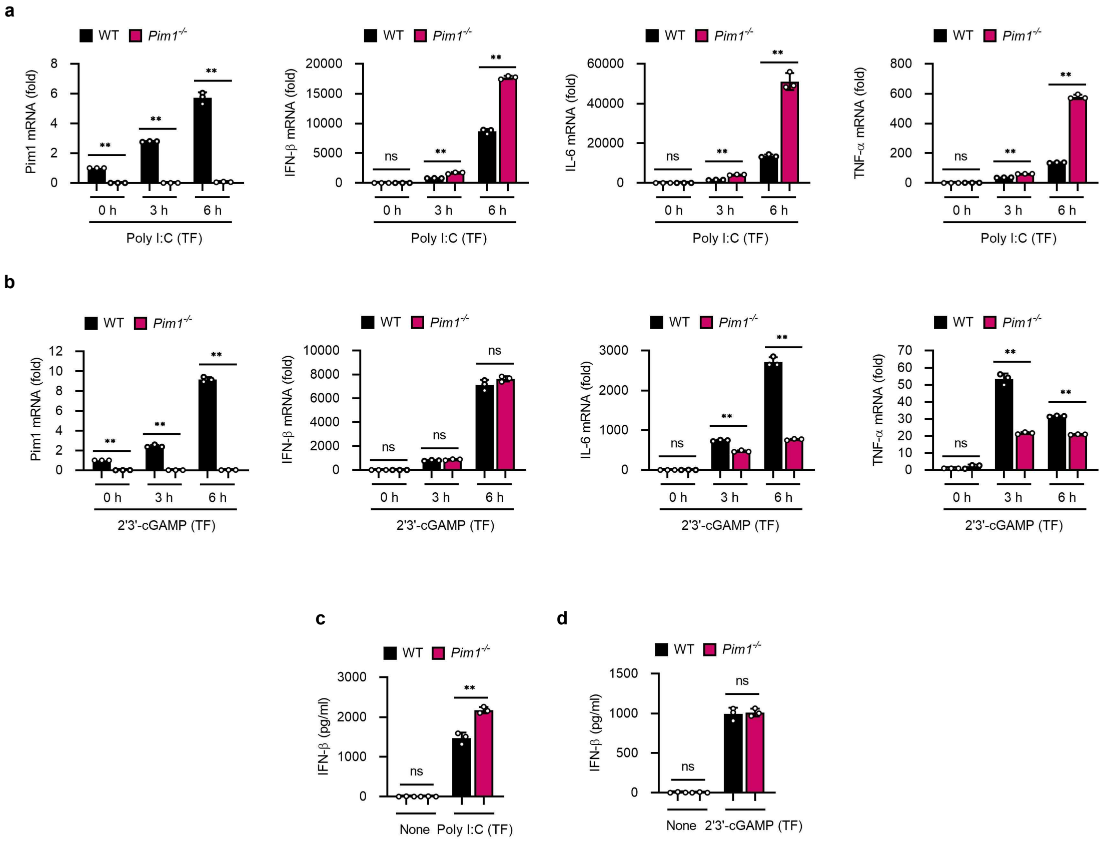

Supplementary Fig. 4. Pim1 differentially regulates RLR- and cGAS-mediated responses in macrophages.

WT or *Pim1*$^{-/-}$ BMDMs were transfected with poly I:C (10 μg ml^{-1}) (**a,c**) or 2’3’-cGAMP (10 μg ml^{-1}) (**b,d**) for up to 6 h. **a,b,** RT-qPCR of Pim1, IFN-β, IL-6 and TNF-α at the indicated times. mRNA values were normalized to β-actin mRNA expression. **c,d**, IFN-β levels in the supernatants at 6 h. All data are presented as mean ± s.d. and represent at least three independent experiments with similar results. $^{**}p < 0.01$, as determined by two-tailed Student’s t-test.

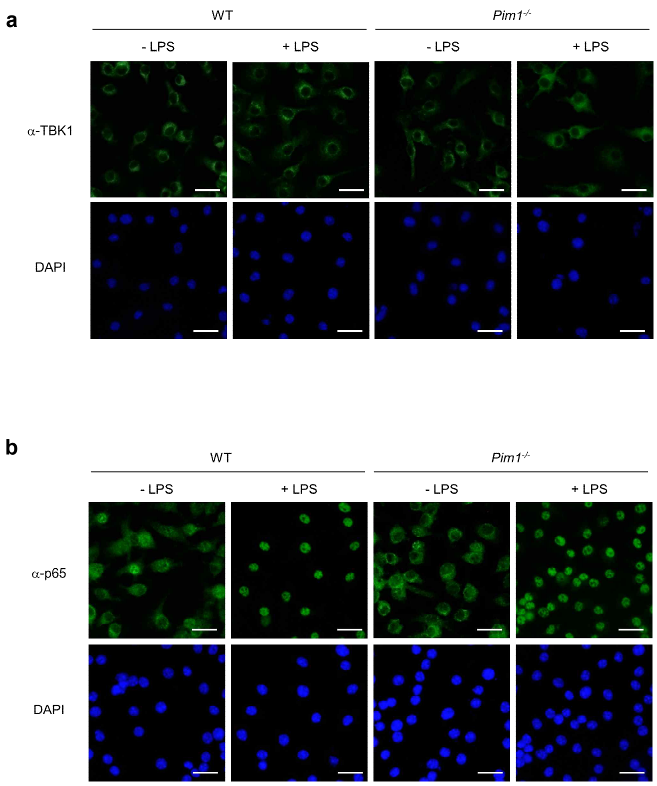

Supplementary Fig. 5. Pim1 does not alter TBK1 localization or NF-κB nuclear translocation.

a,**b**, Immunostaining of TBK1 (**a**) and NF-κB p65 (**b**) in WT or *Pim1*$^{-/-}$ BMDMs treated with LPS (100 ng ml^{-1}) for 1 h. Nuclei were counterstained with DAPI. Scale bars represent 10 μm. Data are representative of at least two independent experiments with similar results.

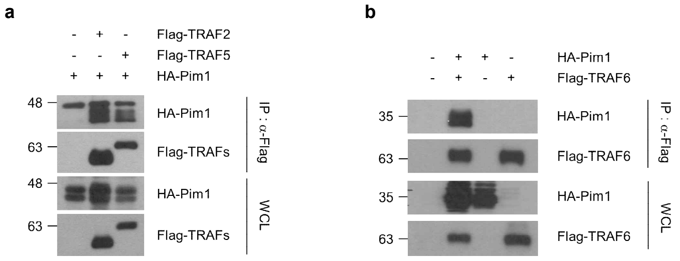

Supplementary Fig. 6. Pim1 co-immunoprecipitates with other TRAFs.

a,b, HEK293T cells were transfected with distinctly tagged Pim1 and TRAF2, TRAF5 (**a**), or TRAF6 (**b**) and then subjected to immunoprecipitation analyses using tag-specific antibodies. All data represent at least two independent experiments with similar results.

Supplementary Table 1. Sequences of qRT-PCR primers

Gene		Sequence (5'→3')
Pim1	F	CGCGACATCAAGGACGAGAACA
	R	CGAATCCACTCTGGAGGACTGT
Pim2	F	CTGCTTCACGATGAGCCGTACA
	R	GAGTAGGACACCTAGTGACCAG
Pim3	F	TGTGGTCTCTGGGTGTACTGCT
	R	GACACCACTCAATAAGCTGCTGG
β-actin	F	CATTGCTGACAGGATGCAGAAGG
	R	TGCTGGAAGGTGGACAGTGAGG
IFN-β	F	GCCTTTGCCATCCAAGAGATGC
	R	ACACTGTCTGCTGGTGGAGTTC
ISG15	F	CATCCTGGTGAGGAACGAAAGG
	R	CTCAGCCAGAACTGGTCTTCGT
ISG54	F	CGAACTACCGTCTGGATGACTG
	R	CTTCAACCAGCGCCATTGCTTG
ISG56	F	TACAGGCTGGAGTGTGCTGAGA
	R	CTCCACTTTCAGAGCCTTCGCA
TNF-α	F	GGTGCCTATGTCTCAGCCTCTT
	R	GCCATAGAACTGATGAGAGGGAG
IL-1β	F	TGGACCTTCCAGGATGAGGACA
	R	GTTCATCTCGGAGCCTGTAGTG
IL-6	F	TACCACTTCACAAGTCGGAGGC
	R	CTGCAAGTGCATCATCGTTGTTC
IL-10	F	CGGGAAGACAATAACTGCACCC
	R	CGGTTAGCAGTATGTTGTCCAGC
